# Supplementary material for: Content-rich biological network constructed by mining PubMed abstracts
Source: BMC Bioinformatics. 2004 Oct 8;5:147. doi: 10.1186/1471-2105-5-147 (PMC528731; doi:10.1186/1471-2105-5-147)
Supplement: Additional File 5 — The original Chilibot query results of the term "long-term potentiation (LTP)" and 22 other terms, limiting the latest references analyzed to the years 1990, 1995, 2000, and 2004. [file 1471-2105-5-147-S5.bz2 › chilibotAdditionalFile5/ltp2004/html/CREB_ACTININ.html]

 


 **CREB** and **ACTININ** 
  
Found 1 abstracts in PubMed,  **1 abstracts were retrieved and analyzed**.  


---

 Search Google  |
 PDF files only 
|  EDU domain only 

---

- J Biol Chem, 2003   **FHL3 is an actin binding protein that regulates alpha **actinin** mediated actin bundling FHL3 localizes to actin stress fibers and enhances cell spreading and stress fiber disassembly.** .
  Four and a half LIM domain FHL proteins are members of the LIM protein superfamily.
  Several FHL proteins function as co activators of CREM **CREB** transcription factors and the androgen receptor.
  FHL3 is highly expressed in skeletal muscle, but its function is unknown.
  FHL3 localized to the nucleus in C2C12 myoblasts and, following integrin engagement, exited the nucleus and localized to actin stress fibers and focal adhesions.
  In mature skeletal muscle FHL3 was found at the Z line.
  Actin was identified as a potential FHL3 binding partner in yeast two hybrid screening of a skeletal muscle library.
  FHL3 complexed with actin both in vitro and in vivo as shown by glutathione S transferase pull down assays and co immunoprecipitation of recombinant and endogenous proteins.
  FHL3 promoted cell spreading and when overexpressed in spread C2C12 cells disrupted actin stress fibers.
  Increased FHL3 expression was detected in highly motile cells migrating into an artificial wound, compared with non motile cells.
  The molecular mechanism by which FHL3 induced actin stress fiber disassembly was demonstrated by low speed actin co sedimentation assays and electron microscopy.
  FHL3 inhibited alpha **actinin** mediated actin bundling.
  These studies reveal FHL3 as a significant regulator of actin cytoskeletal dynamics in skeletal myoblasts.
